# Identification of a novel six autophagy-related genes signature for the prognostic and a miRNA-related autophagy predictor for anti-PD-1 therapy responses in prostate cancer

**DOI:** 10.1186/s12885-020-07725-0

**Published:** 2021-01-05

**Authors:** Lei Wu, Wen Quan, Guojun Yue, Qiong Luo, Dongxu Peng, Ying Pan, Guihai Zhang

**Affiliations:** 1 Department of Oncology, Zhuhai People’s Hospital (Zhuhai Hospital affiliated with Jinan University), Zhuhai, Guangdong Province P. R. China; 2 Zunyi Medical University, Zunyi, Guizhou Province P. R. China; 3 Department of Oncology, Affiliated Zhuhai Hospital, Southern Medical University, Zhuhai, Guangdong Province P. R. China

**Keywords:** Autophagy, miRNA, Prostate cancer, PD-1

## Abstract

**Background:**

Autophagy is a highly conserved homeostatic process in the human body that is responsible for the elimination of aggregated proteins and damaged organelles. Several autophagy-related genes (ARGs) contribute to the process of tumorigenesis and metastasis of prostate cancer (PCa). Also, miRNAs have been proven to modulate autophagy by targeting some ARGs. However, their potential role in PCa still remains unclear.

**Methods:**

An univariate Cox proportional regression model was used to identify 17 ARGs associated with the overall survival (OS) of PCa. Then, a multivariate Cox proportional regression model was used to construct a 6 autophagy-related prognostic genes signature. Patients were divided into low-risk group and high-risk group using the median risk score as a cutoff value. High-risk patients had shorter OS than low-risk patients. Furthermore, the signature was validated by ROC curves. Regarding mRNA and miRNA, 12 differentially expressed miRNAs (DEMs) and 1073 differentially expressed genes (DEGs) were detected via the GEO database. We found that miR-205, one of the DEMs, was negatively regulated the expression of ARG (NKX2–3). Based on STRING analysis results, we found that the NKX2–3 was moderately related to the part of genes among the 6 autophagy-related genes prognostic signature. Further, NKX 2–3 was significantly correlated with OS and some clinical parameters of PCa by cBioProtal. By gene set enrichment analysis (GSEA). Lastly, we demonstrated that the association between NKX2–3 and tumor mutation burden (TMB) and PDCD1 (programmed cell death 1) of PCa.

**Results:**

We identified that the six ARGs expression patterns are independent predictors of OS in PCa patients. Furthermore, our results suggest that ARGs and miRNAs are inter-related. MiR-205 was negatively regulated the expression of ARG (NKX2–3). Further analysis demonstrated that NKX2–3 may be a potential biomarker for predicting the efficacy of anti-PD-1 therapy in PCa.

**Conclusions:**

The current study may offer a novel autophagy-related prognostic signature and may identify a promising miRNA-ARG pathway for predicting the efficacy of anti-PD-1 therapy in PCa.

## Background

Prostate cancer is one of the most common malignancies in men. The incidence of prostate cancer has been increasing worldwide in recent years [1]. Many patients within the early stage have a good prognosis after several effective therapies. Nevertheless, some progressive prostate cancer patients with advanced-stage are more resistant to the conventional treatments. As a result, these patients have a poor prognosis and high cancer-related mortality [2]. Therefore, it is imperative to develop some novel and effective therapeutic strategies for prostate cancer patients.

Autophagy is a major physiological process responsible for the elimination of aggregated proteins and damaged organelles [3]. Increasing studies have shown that autophagy is closely associated with various cancers, including prostate cancer [4–6]. Nevertheless, the mechanism of autophagy in tumorigenesis is multifaceted [7]. Previous experiments have demonstrated that autophagy has been considered as a double-edged sword in carcinogenesis, which either promotes or inhibits the development of cancers in different stages [8]. Before a tumor develops, autophagy is thought to prevent cancer development in non-cancerous cells by eradicating damaged organelles and oncogenic protein substrates [9, 10]. However, once the cancer has developed, autophagy protects tumor cells against stress as an adaptive response, and thus promotes tumor progression [11, 12]. It is obvious that autophagy is a promising therapeutic target for cancer therapy. Thus, it is essential to discover the autophagy-related mechanism in cancer and its therapeutic targeting in tumor microenvironment.

MicroRNAs (miRNAs) are a family of endogenous non-coding RNAs that negatively control gene expression at the post-transcriptional level [13, 14]. Aberrant expression of miRNA is essential for the occurrence and development of human malignant tumors, because they act as both tumor suppressors and oncogenes [15]. Numerous studies have demonstrated that some miRNAs significantly modulate the biological behaviors of prostate cancer [16]. For instance, miR-29b inhibits prostate tumor growth by targeting Bim [17]. MiR-135a regulates apoptosis in prostate cancer through the inhibition of STAT6 [18]. Downregulated expression of miR-139-5p promotes prostate cancer progression by targeting SOX5 [19]. Further, miRNAs also can be invoked as potential prognostic biomarkers in prostate cancer [20–22].

Numerous studies have confirmed that many miRNAs can affect the biological behaviors of multiple tumor tissues by regulating autophagy-related genes or pathways [23–26]. However, the role of miRNAs and autophagy in tumorigenesis have analyzed a limited number of ARGs and miRNAs in prostate cancer in most of the previous studies. Hence, the prognostic value of ARGs and the regulatory mechanism of ARGs and miRNAs have not been clearly and completely realized in prostate cancer. Therefore, it will be useful to further understand the regulation mechanisms between autophagy and miRNAs in prostate cancer.

In our study, we presented NKX2–3 as one of the prognostic biomarkers for PCa and founded that NKX2–3 relied to the autophagy is highly expressed in PCa tissues. Meanwhile, NKX2–3 was significantly related to the clinical parameters and OS of prostate cancer patients. Additionally, miR-205 has decreased expression in PCa samples, compared with normal prostate samples. Herein, we characterize that NKX2–3 is up-regulated by miR-205. MiR-205/NKX2–3 regulation pathway may have a modulatory effect on autophagy in PCa. Finally, since the expression of NKX2–3 was specifically correlated with PD-1 and TMB, NKX2–3 may be used as a biomarker for the prediction of PD-1 efficacy in PCa.

## Methods

### Acquisition of prostate cancer datasets

The transcriptome expression profiles and corresponding clinical information of prostate cancer were obtained from the TCGA database. The expression data was HTSeq-FPKM type, containing 499 prostate cancer tissues and 52 adjacent non-tumor samples. GSE36802 and GSE69223 datasets were obtained from the Gene Expression Omnibus (GEO). Microarray data of GSE36802 and GSE69223 were on account of GPL8786 and GPL570 platforms, respectively. The GSE36802 dataset contained 42 samples including 21 prostate cancer tissues and 21 non-tumorous tissues. The GSE69223 dataset contained 30 samples including 15 prostate cancer tissues and 15 non-tumorous tissues. DEMs and DEGs were identified by comparing prostate cancer tissues with noncancerous prostate tissues using the limma package of R software (Version 3.6.1). |logFC| > 1 and adjust *P* value < 0.05 were set as the cut-off values.

### Human autophagy related genes

The 232 autophagy-related genes were obtained from the Human Autophagy Database (HADb, http://autophagy.lu/clustering/index.html). These genes had been described involved in the autophagy process based on literature [27].

### Gene ontology and pathway enrichment analysis of the DEMs and DEGs

Gene ontology (GO) and pathway analysis were performed using the functional enrichment analysis tool (FunRich v3.1.3), which included cellular component (CC), molecular function (MF), and biological process (BP), and a biological pathways pathway analysis of the DEMs and DEGs [28, 29]. *P* < 0.05 was set as the cutoff criterion.

### Gene set enrichment analysis (GSEA)

GSEA was carried out to analyze the biological pathway in prostate cancer stratified by the median expression of NKX2–3. The detailed process follows the recommended protocol from the Broad Institute Gene Set Enrichment Analysis website [30]. The GSEA was performed using the GSEA v4.0.3 software. The gene sets were adopted from The Molecular Signatures Database within the Hallmark gene sets [31]. NOM *p*-value < 0.05 and FDR q-value < 0.05 were recognized as statistically significant.

### Statistical analysis

All statistics were performed with the R software (Version 3.6.1). × 2 test and t-test were used to check for categorical variables and continuous variables, respectively. Based on the median value of risk score, Kaplan-Meier curves were plotted and a log-rank test was used to check the significant difference in overall survival between high-risk and low-risk groups. The receiver operating characteristic (ROC) analysis was used to examine the sensitivity and specificity of survival prediction using the gene signature risk score. An area under the ROC curve (AUC) served as an indicator of prognostic accuracy. Pearson correlations were used to examine correlations between variables. *P* < 0.05 was considered statistically significant for all the analyses.

## Results

### Identification of an autophagy-related risk signature for the prognosis of prostate cancer

**Fig. 1:**
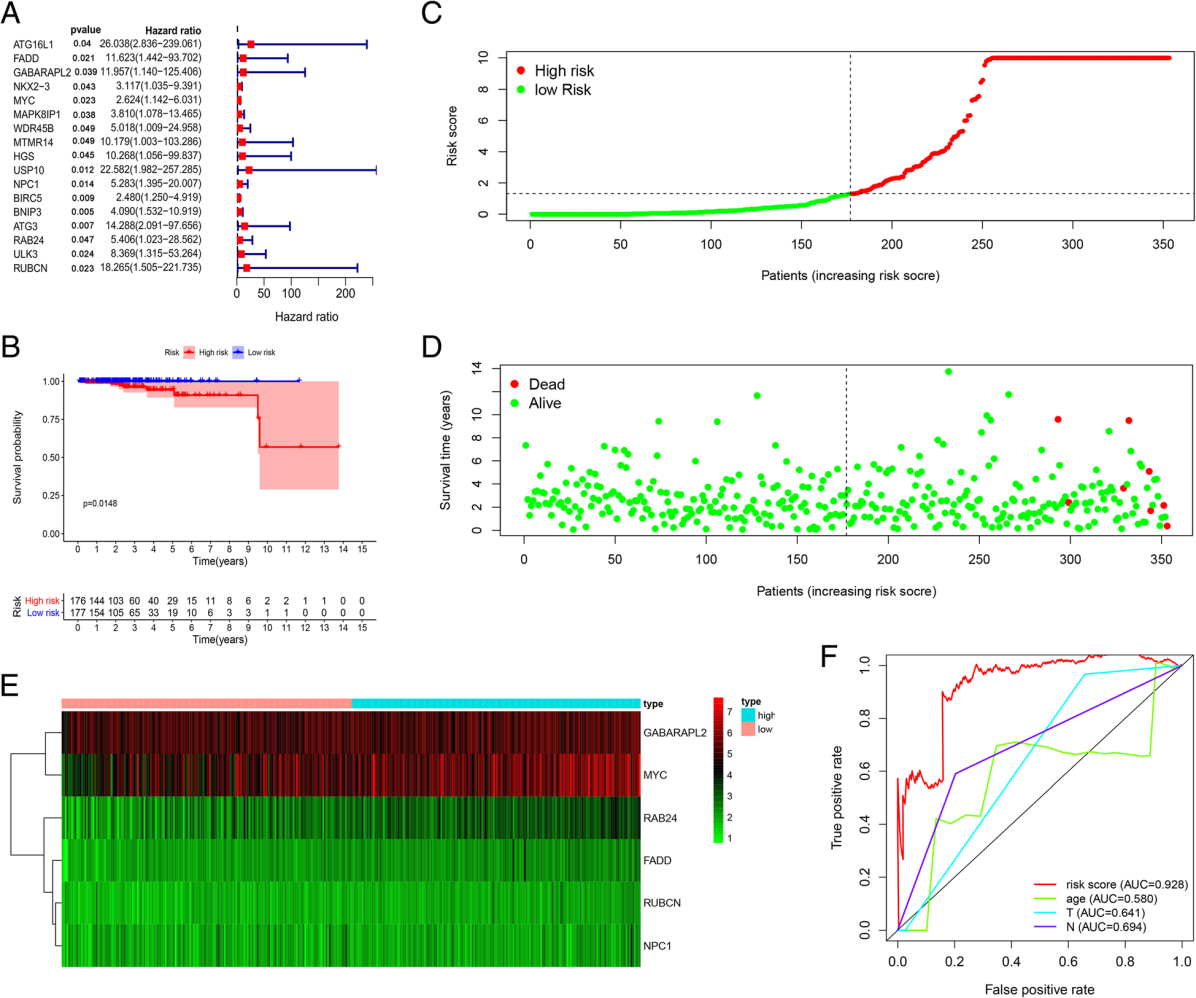
Identification of potential prognostic markers for prostate cancer. **a** 17 autophagy genes that significantly relate to OS were certified utilizing univariate Cox regression. **b** Difference of overall survival between low-risk and high-risk groups. **c** and **d** The distribution of risk score and patient’s survival time, as well as survival status for prostate cancer. The black dotted line is the optimum cutoff dividing patients into low risk and high risk groups. **e** Heatmap of high-risk and low-risk patients expressing the 6 genes. **f** The 3-year survival ROC of the six-gene signature and classical clinicopathologic parameters in the TCGA dataset. The figures were created using R software v3.6.1

The prognostic value of ARGs was performed by univariate COX regression in 499 prostate tumor samples in the TCGA database. Seventeen genes (ATG16L1, FADD, GABARAPL2, NKX2–3, MYC, MAPK8IP1, WDR45B, MTMR14, HGS, USP10, NPC1, BIRC5, BNIP3, ATG3, RAB24, ULK3 and RUBCN) were identified to be significantly correlated with OS in prostate tumor samples. Among the 17 genes, all the genes were identified as risk factors (*p* < 0.05, HR > 1, Fig. 1a). And then a multivariate Cox regression was performed to develop the following autophagy-related risk signature related to the survival of PCa patients. The calculation formula of risk score is as follows [32]: Risk score = (FADD expression× 2.341572) + (GABARAPL2 expression× 4.34965) + (MYC expression× 2.430837) + (RAB24 expression× 3.570068) + (RUBCN expression× 4.857469) + (NPC1 expression× 1.739033). Then patients were divided into low-risk group (*n* = 177) and high-risk group (*n* = 176) using the median risk score as a cutoff value. Our data showed that the survival time of high risk group is significantly shorter than the low risk group (Fig. 1b). Risk genes showed significant expression patterns according to the risk value (Fig. 1c) and all of the deaths occurred in the high-risk group (Fig. 1d). The heatmap of the 6 ARGs expression levels in the TCGA dataset, high-risk group expressed higher levels of risky genes (Fig. 1e). ROC curves for 3-year overall survival were performed to evaluate the predictive power of the six-gene risk signature (Fig. 1f). The 3-year AUC of our signature was 0.928, which was obviously higher than that of age (AUC = 0.580), tumor T stage (AUC = 0.641), and tumor N stage (AUC = 0.694).

### The correlation between the autophagy-related risk signature and clinical factors in prostate cancer

**Fig. 2:**
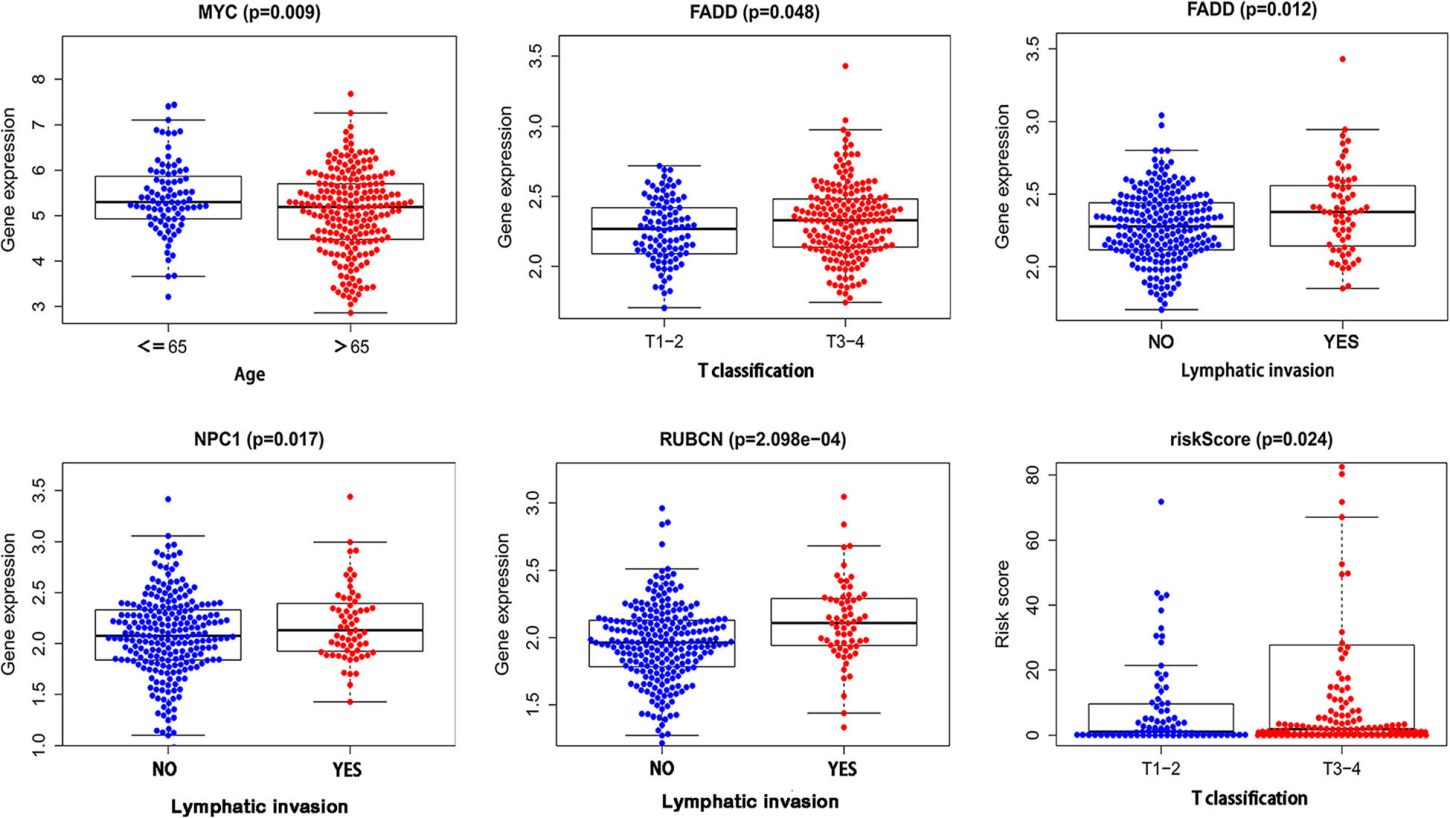
The significance analysis of predicted gene signature association with clinical factors and the association between risk score and clinical factors. The figures were created using R software v3.6.1

An analysis was applied to compare the correlation between predicted 6-gene signature and conventional clinical factors of prostate cancer, Results showed that MYC expression was correlated with patients’ age (*P* = 0.009). FADD expression was correlated with both T classification (P = 0.009) and lymphatic invasion (*P* = 0.012). NPC1 expression was associated with lymphatic invasion (*P* = 0.017). RUBCN expression was correlated with lymphatic invasion (*P* = 2.098e-04). We further analyzed the correlation between the risk score and these clinical factors, we found that T classification was correlated with the risk score (*P* = 0.024) (Fig. 2).

### Identification of DEMs and DEGs in prostate cancer

**Fig. 3:**
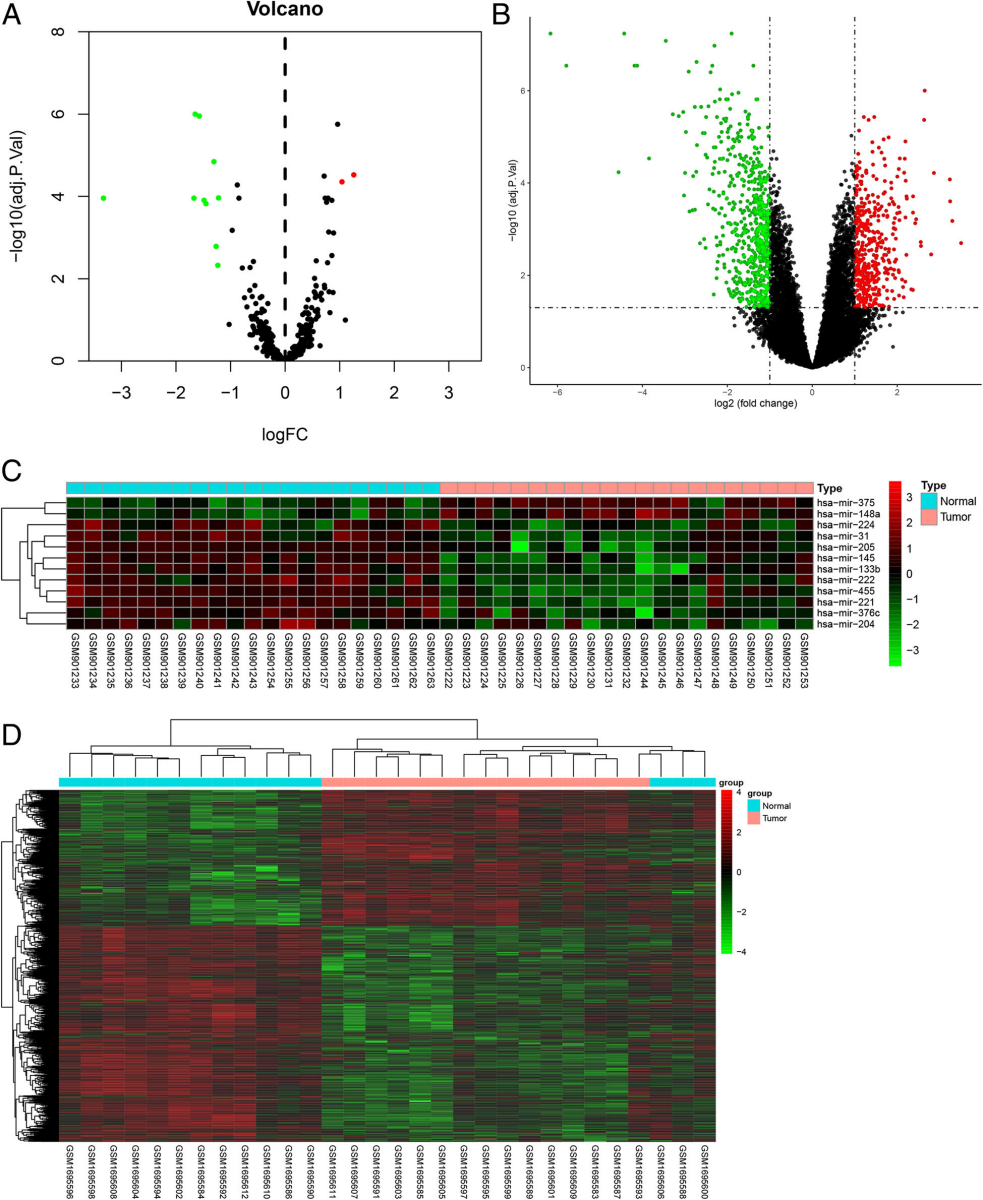
DEMs and DEGs between prostate cancer and normal prostate tissues. **a** and **b** The volcano plot for the 12 DEMs and 1073 DEGs from the GEO. Green means downregulated DEMs and DEGs; red means upregulated DEMs and DEGs. **c** and **d** Hierarchical clustering of DEMs and DEGs expression levels. The figures were created using R software v3.6.1

There are 21 pairs of PCa samples and matched adjacent non-tumor prostate samples were collected and processed for microRNA detection, and mRNA expression analysis was performed by using 30 matched malignant and normal prostate tissue samples from 15 prostate cancer patients. All the samples were obtained from GEO. Results showed that a total of 12 DEMs and 1073 DEGs were detected. Considered as the criteria of |log FC| > 1 and adjust *P* value < 0.05, we finally identified 2 up-regulated and 10 down-regulated DEMs (Fig.3a and c). Meanwhile, 413 up-regulated and 660 down-regulated DEGs were extracted (Fig. 3b and d).

### Enrichment analysis of the DEMs and DEGs

**Fig. 4:**
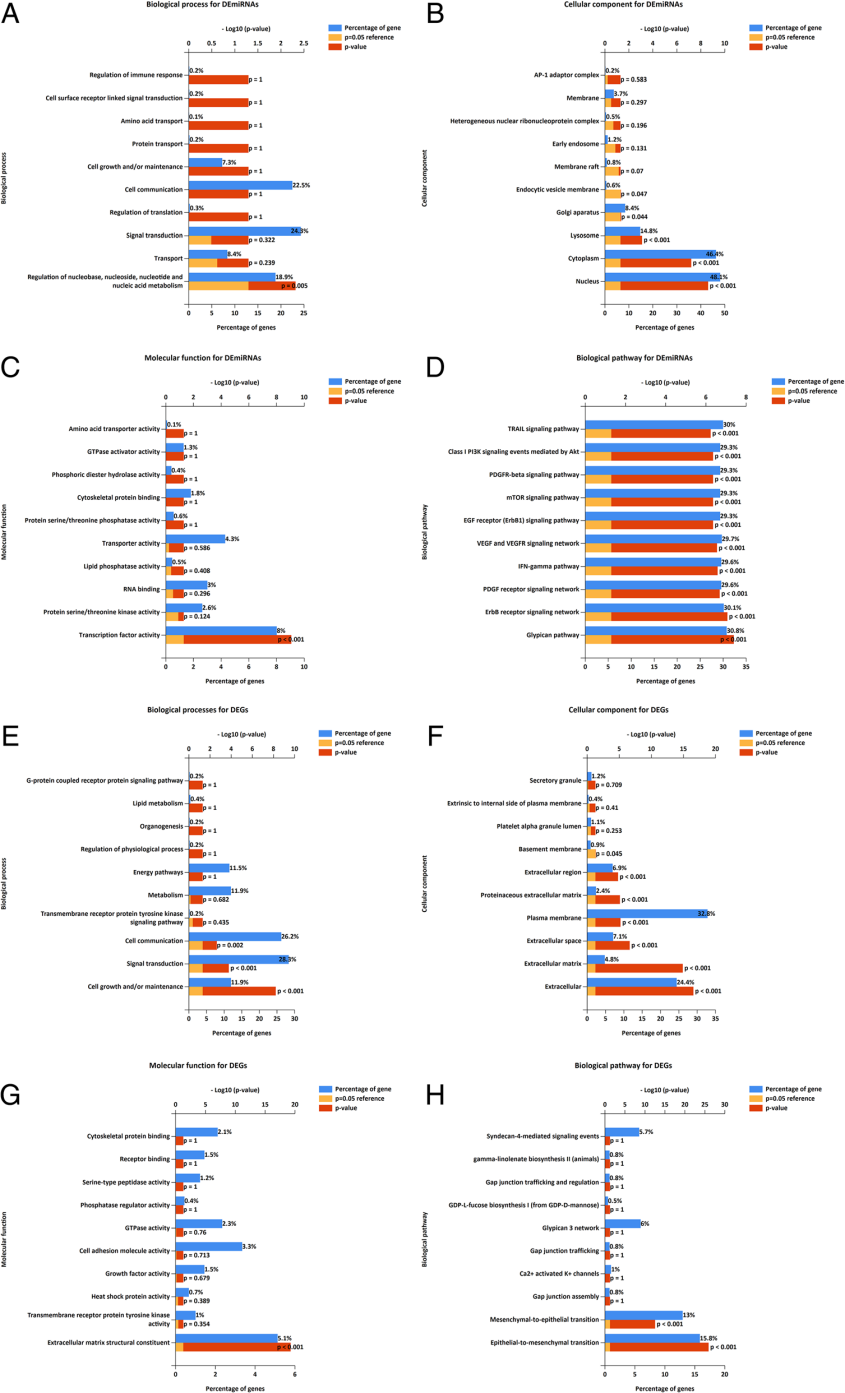
Gene Ontology and and biological pathway enrichment analyses of the DEMs and DEGs in prostate cancer. **a** BP pathways, **b** CC pathways, **c** MF pathways, and **d** Biological pathways of the DEMs in prostate cancer; **e** BP pathways, **f** CC pathways. **g** MF pathways. **h** Biological pathways of the DEGs in prostate cancer. The figures were created using R software v3.6.1

To improve our understanding of the biological information of the 12 DEMs in prostate cancer, we performed GO annotation and biological pathway analyses by using the software of FunRich. Regarding BP, the DEmiRNAs were significantly enriched in regulation of nucleic acid metabolism,, nucleotide, nucleoside, and nucleobase (Fig. 4a). Regarding CC, the DEmiRNAs were significantly enriched in nucleus, cytoplasm, lysosome, golgi apparatus, and endocytic vesicle membrane (Fig. 4b). In addition, the significantly enriched GO terms in MF was transcription factor activity (Fig. 4c). As shown in Fig. 4d, the pathways of biological processes were TRAIL signaling pathway, Class I PI3K signaling events mediated by Akt, PDGFR-beta pathway, mTOR signaling pathway, EGF receptor pathway, VEGF and VEGFR network, IFN-gamma pathway, ErbB receptor signaling network, Glypican pathway, and PDGF receptor signaling network. Alike, all 1073 DEGs were also uploaded to the FunRich, the results of GO analysis indicated that 1) for BP, DEGs were significantly enriched in cell communication, signal transduction, and cell growth and/or maintenance; 2) for CC, DEGs were particularly enriched in the basement membrane, extracellular region, proteinaceous extracellular matrix, plasma membrane, extracellular space, extracellular matrix, and extracellular. 3) for MF, DEGs was only enriched in extracellular matrix structural constituent (Fig. 4e-g). Additionally, biological pathway analysis showed these DEGs were mostly enriched in mesenchymal-to-epithelial transition and epithelial-to-mesenchymal transition (Fig. 4h).

### miRNA–mRNA network

**Fig. 5:**
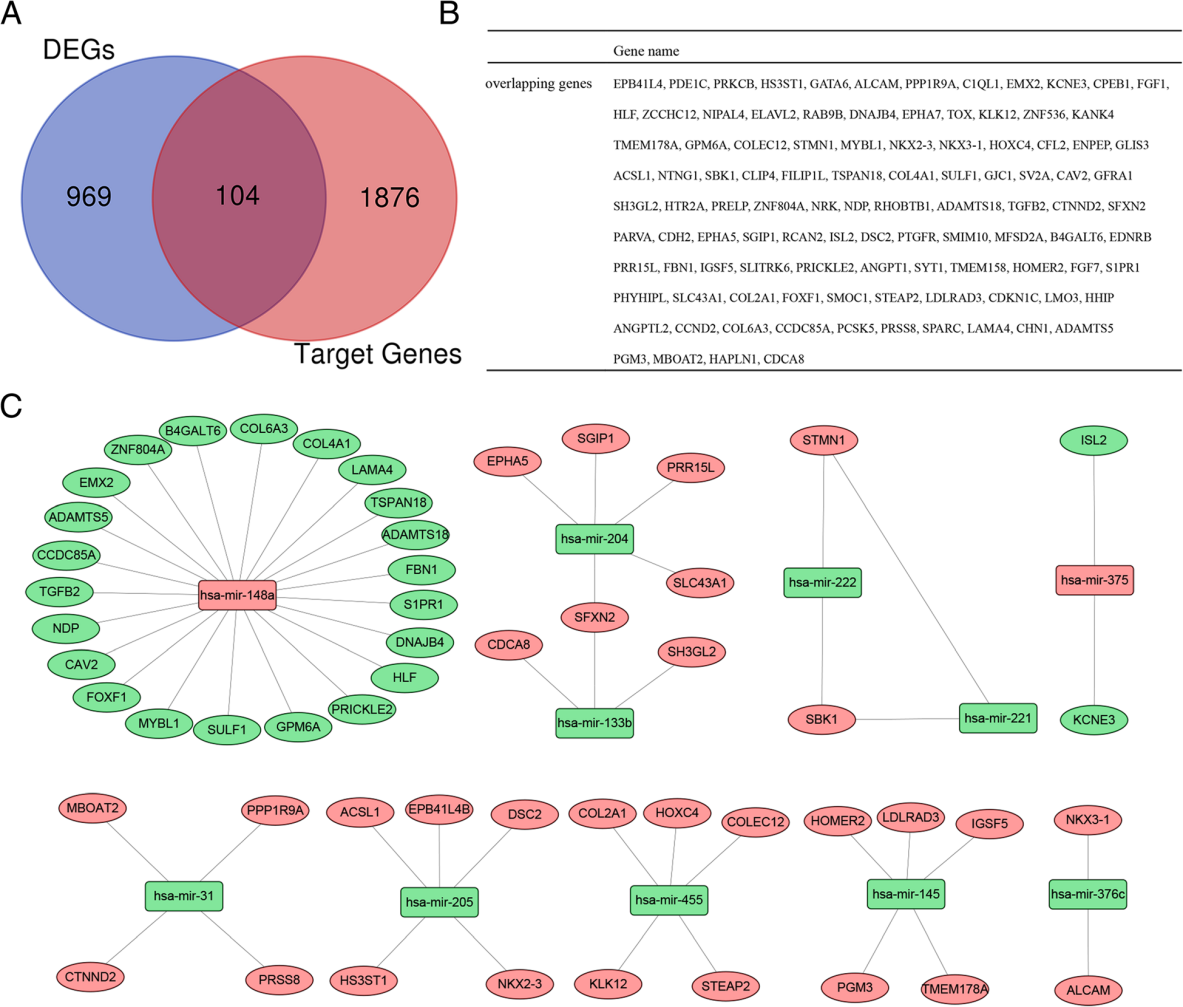
A regulatory network of the overlapping genes and their target miRNAs. **a** Venn diagram of DEGs overlapping with DEMs target genes. **b** Names of 104 overlapping genes. **c** Circle nodes indicate hub genes, rectangle nodes indicate DEMs. Red nodes represent up-regulated and green nodes represent down-regulated. The figures were created using R software v3.6.1

FunRich software was utilized to predict potential target genes from DEMs. All the 12 DEMs were inducted into the FunRich software. There were 1980 target genes found. Then, we further assessed the intersection of 1980 target genes and 1073 DEGs, and obtained 104 overlapping genes for subsequent analysis (Fig. 5a and b). The network results of DEMs and overlapping genes were calculated using FunRich software, and the results were visualized in Cytoscape software (Fig. 5c). Notably, hsa-miR-148a targeted 22 genes, including ADAMTS18, ADAMTS5, CAV2, CCDC85A, COL6A3, DNAJB4, EMX2, FBN1, FOXF1, GPM6A, HLF, MYBL1, NDP, PRICKLE2, S1PR1, SULF1, TSPAN18, ZNF804A, B4GALT6, COL4A1, LAMA4, TGFB2; hsa-miR-133b targeted 3 genes, including SH3GL2, SFXN2, CDCA8; hsa-miR-204 targeted 5 genes, including SLC43A1, SGIP1, PRR15L, SFXN2, EPHA5; hsa-miR-222 targeted 2 genes, including STMN1 and SBK1; hsa-miR-221 targeted 2 genes, including STMN1 and SBK1; hsa-miR-31 targeted 4 genes, including MBOAT2, PPP1R9A, CTNND2, PRSS8; hsa-miR-205 targeted 5 genes, including DSC2, NKX2–3, HS3ST1, ACSL1, EPB41L4B; hsa-miR-455 targeted 5 genes, including COL2A1, HOXC4, COLEC12, KLK12, STEAP2; hsa-miR-145 targeted 5 genes, including HOMER2, IGSF5, LDLRAD3, PGM3, TMEM178A; hsa-miR-375 targeted 2 genes, including ISL2 and KCNE3; hsa-miR-376c targeted 2 genes, including ALCAM and NKX3–1; These results indicated that in all the relationships between DEMs and DEGs, miR-205 can specifically regulate the expression of autophagy-related gene NKX2–3.

### Construction of a interaction network between NKX2–3 and autophagy-related risk signature

**Fig. 6:**
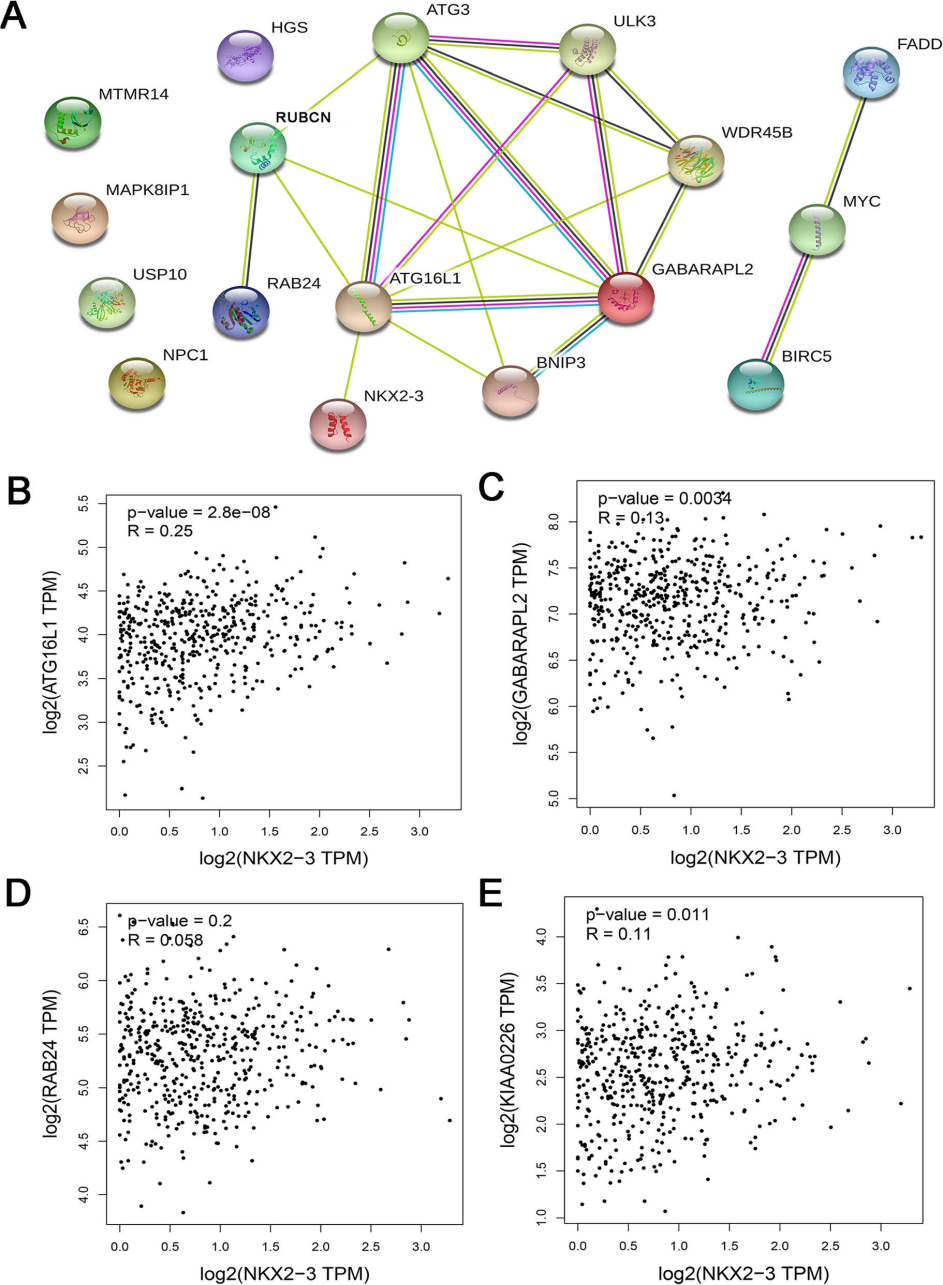
Construction of a interaction network between NKX2–3 and autophagy-related risk signature. **a** The protein interaction network was constructed using STRING v11. Association between NKX2–3 expression and **b** ATG16L1, **c** GABARAPL2, **d** RAB24, **e** RUBCN. The figures were created using R software v3.6.1

STRING database was performed to diagram a network of interacting relationships among the 17 ARGs obtained in Fig. 1a. As shown in Fig. 6a, ATG16L1 can act as a bridge node between NKX2–3 and three of the six genes risk signature (GABARAPL2, RUBCN, RAB24). In other words, we speculated whether NKX2–3 can affect above risk signature through regulating GABARAPL2, RUBCN, and RAB24 expression. To validate this network further, Pearson correlation analysis was used to determine the correlation between NKX2–3 expression and the three genes associated with the risk signature. The results indicated that significant positive correlations were observed between NKX2–3 and ATG16L1, GABARAPL2, KIAA0226 (RUBCN) respectively (Fig. 6b-e).

### Identification of differentially expressed ARGs in prostate cancer

**Fig. 7:**
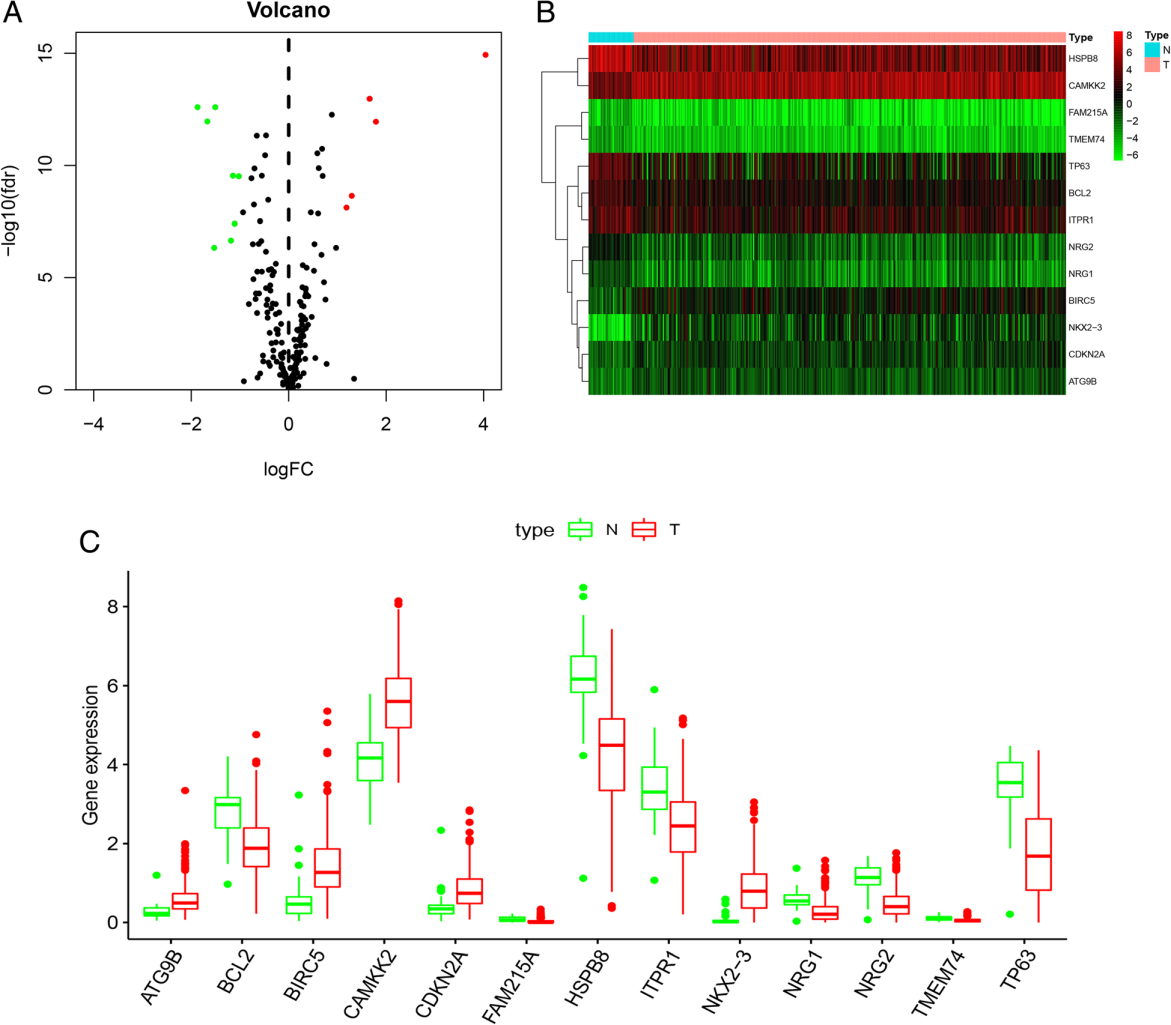
Differentially expressed ARGs between tumor tissues and non-tumor tissues of PCa patoents. **a** Volcano plot of 232 ARGs from the TCGA database. Red dots and green dots represent the upregulated and downregulated ARGs, respectively. **b** Heatmap of differentially expressed ARGs expression levels. **c** The expression patterns of 13 ARGs in prostate cancer and non-tumor tissues. Each red dot represents a distinct tumor sample and green a non-tumor sample. The figures were created using R software v3.6.1

Altogether clinical data and transcriptome expression profiles were downloaded from TCGA. Expression values of 232 ARGs were extracted, we finally identified 5 up-regulated and 8 down-regulated ARGs under cut-off criteria of FDR < 0.05 and |logFC| > 1 (Fig. 7a and b). Furthermore, as presented in Fig. 7c, box plots displayed expression patterns of 8 down-regulated genes (NRG2, BCL2, NRG1, HSPB8, FAM215A, TMEM74, TP63, and ITPR1) and 5 up-regulated genes (NKX2–3, CDKN2A, BIRC5, CAMKK2, and ATG9B).

### Survival analysis of NKX2–3 and clinical parameters in patients with prostate cancer

**Fig. 8:**
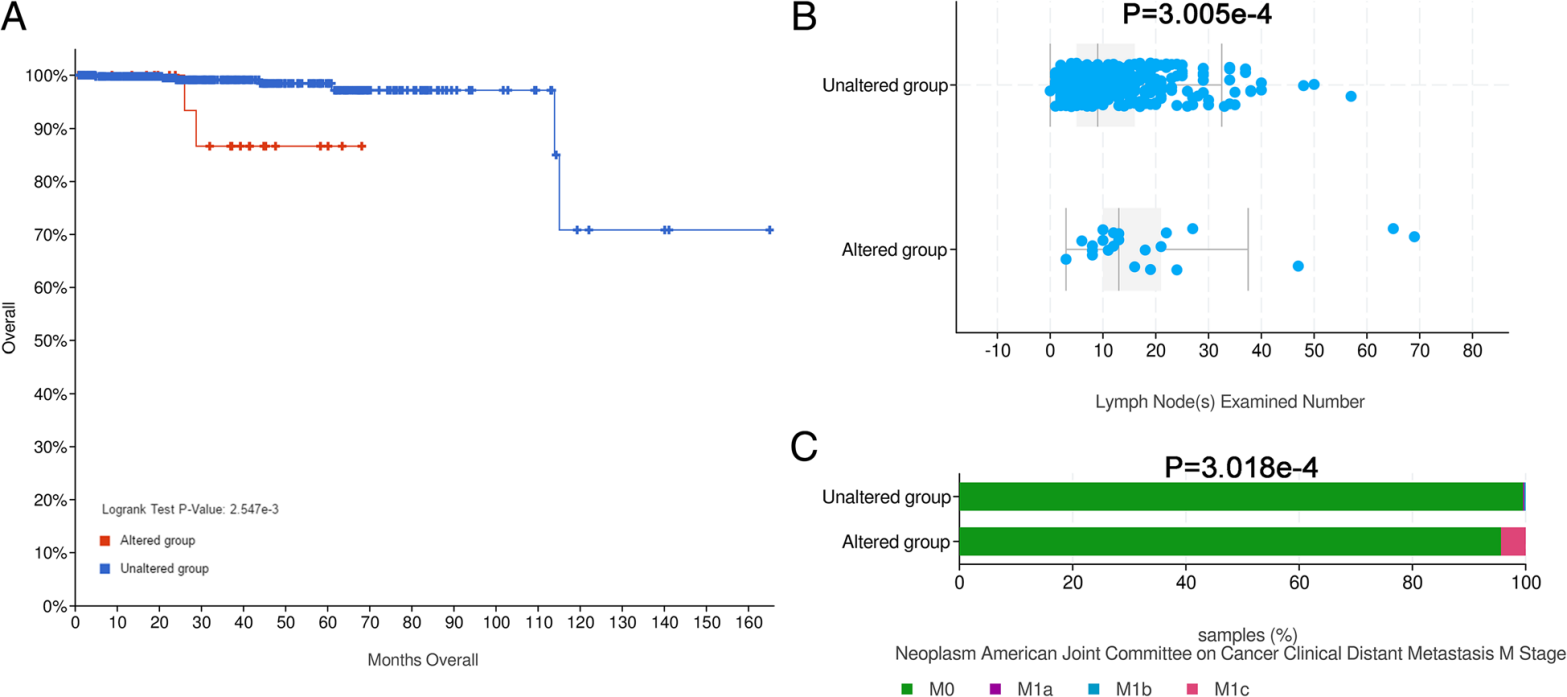
The significance analysis of NKX 2–3 association with overall survival and the association between NKX 2–3 and clinical parameters. **a** Overall survival Kaplan-Meier plot of NKX2–3 from TCGA datasets termed Prostate Cancer (TCGA, Firehose Legacy). The Kaplan-Meier survival curves showed the significant prognostic value of NKX2–3 alteration regarding survival. Red line represents cases with alterations. Blue line represents cases without alterations. **b** and **c** Association between NKX2–3 and clinical parameters in patients with prostate cancer. The figures were created using R software v3.6.1

Based on the results described above, we found that the abnormally high expression of autophagy-related gene NKX2–3 in prostate cancer not only serves as a prognostic risk factor for prostate cancer patients, but also has a specific regulatory relationship with miR-205. Therefore, we further analyzed the clinical value of NKX2–3. The cBioPortal is an open-access resource for interactive exploration of multidimensional cancer genomics data sets and provides comprehensive analyses [33, 34]. The NKX2–3 overall survival analysis using cBioPortal datasets termed prostate cancer (TCGA, Firehose Legacy). Our data showed that the altered group had a shorter overall survival time than the unaltered group (Fig. 8a). Then, we further analyzed the relationship between NKX2–3 and some common clinical parameters. The results showed that the lymph nodes examined number and distant metastasis rate were significantly increased in altered group, compared with the corresponding unaltered group (Fig. 8b and c).

### GSEA by NKX2–3 stratification in prostate cancer

**Fig. 9:**
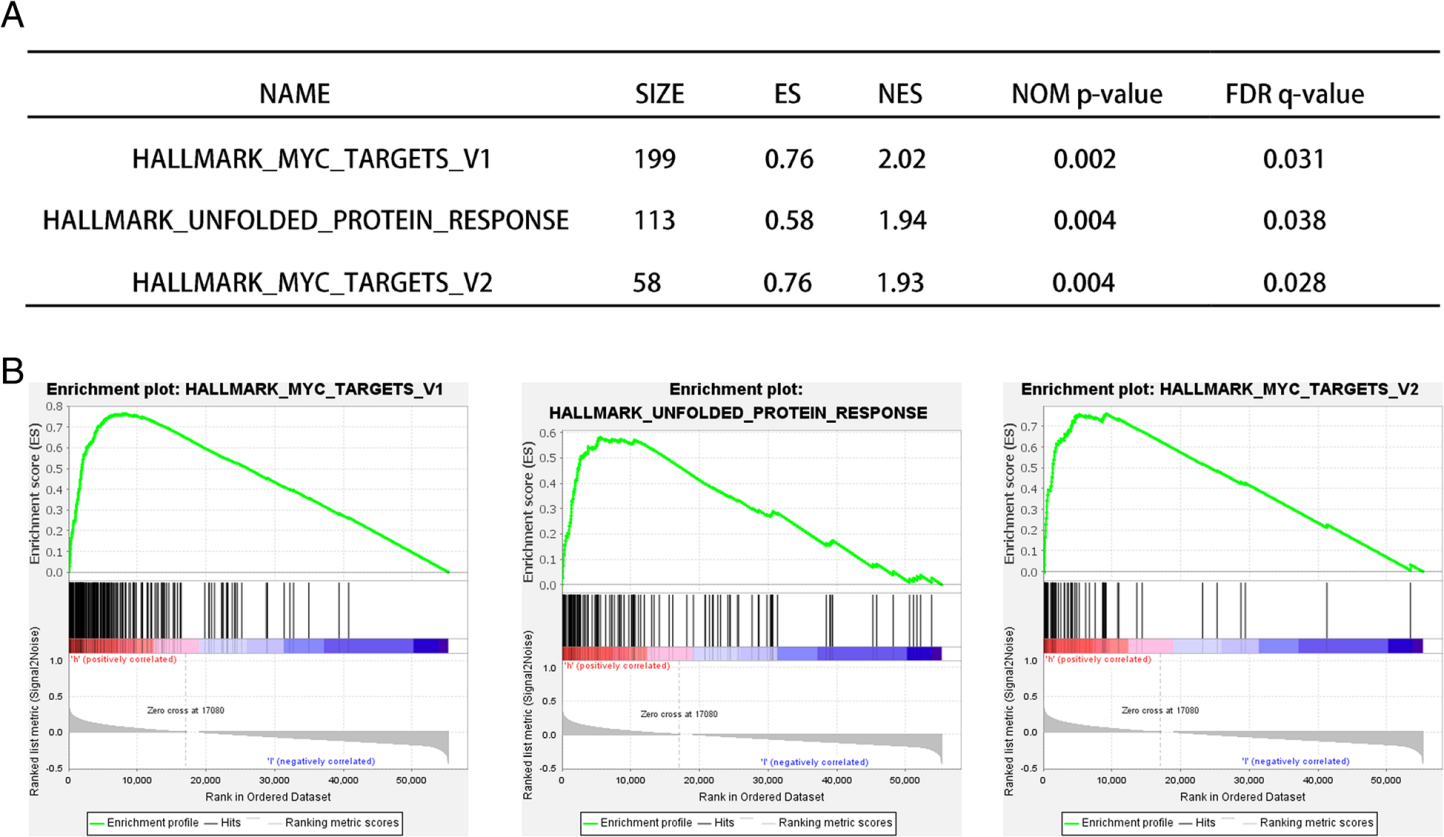
A single gene GSEA in prostate cancer stratified by median NKX2–3 expression. **a** Summary of GSEA results. **b** Individual gene set enrichment plots of GSEA results by high NKX2–3 expression. The figures were created using GSEA software v4.0.3

To explore the mechanism of NKX2–3, patients were separated into high/low expression groups based on the median expression of NKX2–3 and then were subjected to GSEA. The GSEA analysis revealed that the high-expression group of NKX2–3 has strikingly upregulated genes enriched in Myc Targets V1, Unfolded Protein Response and Myc Targets V2 (Fig. 9).

### NKX2–3 may serve as a potential predictor for the efficacy of anti-PD-1 therapy in prostate cancer

**Fig. 10:**
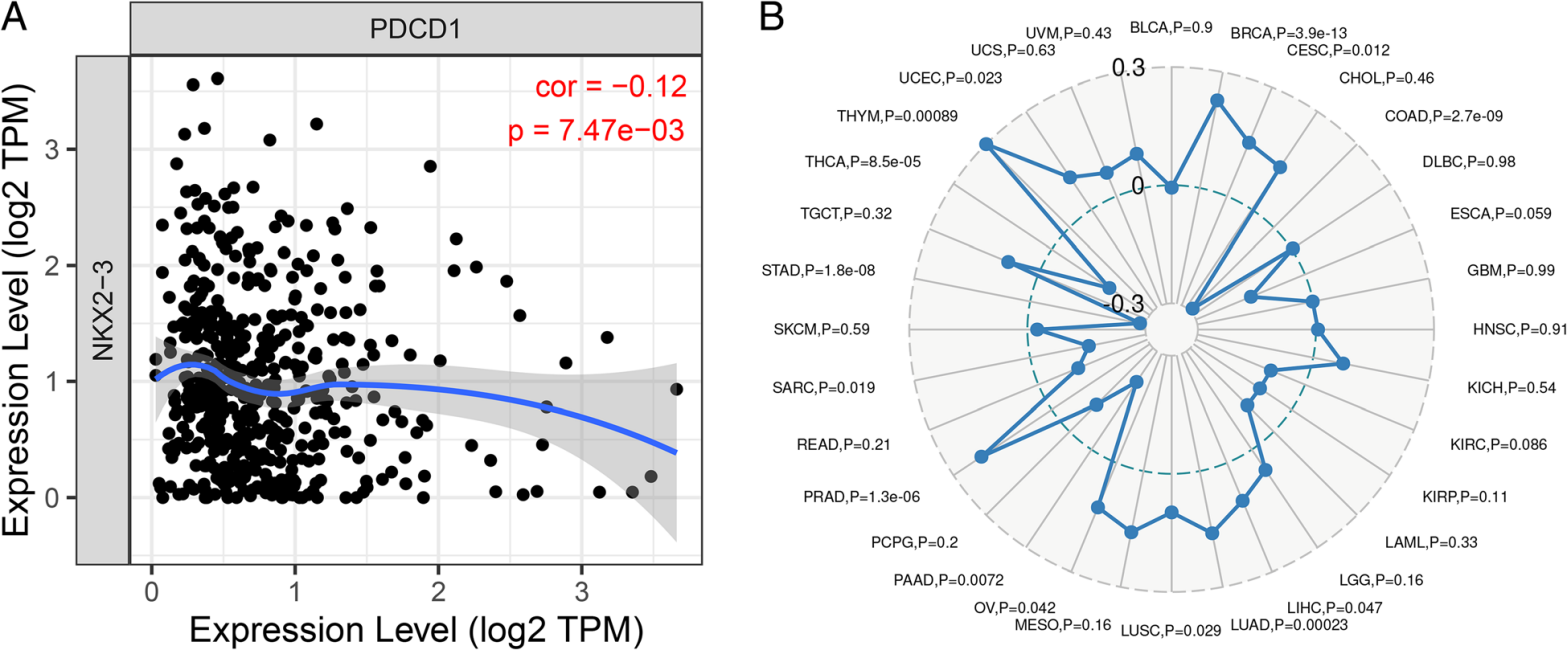
Correlation analysis of NKX2–3 expression with PD-1 and TMB in prostate cancer. **a** Identification of the correlation between the NKX2–3 and PD-1 in prostate cancer. **b** Radar plot of NKX2–3 in relation to TMB in 32 cancer types. Green dotted line benchmarks the correlation of the NKX2–3 and TMB. The figures were created using R software v3.6.1

To further test the correlation of NKX2–3 with immunotherapy response of prostate cancer, we further analyzed the relationship between NKX2–3 and PD-1 (programmed cell death 1, PDCD1). The relevance between the expression of NKX2–3 and PD-1 was analyzed by TIMER, which is a comprehensive resource for systematical analysis of immune infiltrates across diverse cancer types [35, 36]. Our data showed that as the expression of NKX2–3 increased, the expression of PD-1 decreased, thus NKX2–3 was negatively correlated with PD-1 in prostate cancer (Fig. 10a). Furthermore, as shown in Fig. 10b, a positive correlation was detected between NKX2–3 expression and the TMB (*P* < 0.05; (tumor mutation burden) of prostate cancer (*P* = 1.3e-06).

## Discussion

Prostate cancer is a major lethal cancer in men worldwide. Thus, it is urgent to identify reliable prognostic biomarkers to improve the clinical treatment of prostate cancer patients. Bioinformatics prediction signatures have recently been applied to develop potential non-coding RNA and mRNA biomarkers for prostate cancer [37, 38]. Furthermore, autophagy is closely connected with tumorigenesis and therapy [39–41]. Exploration of autophagy mechanism opens new horizons for prostate cancer. However, most related studies of ARGs only focused on a signal gene. Our research used high-throughput expression profiling to capture the genes necessary for prostate cancer from the perspective of autophagy. Then, we selected more than one key prognostic ARGs, all of them may be potential therapeutic targets in prostate cancer. We further leveraged the complementary value of DEMs and ARGs and showed that miR-205 could specifically bind the autophagy-related gene NKX2–3, and NKX2–3 may be a potential predictive marker for the efficacy of anti-PD-1 therapy in prostate cancer. This integrated study of multiple databases opens up a new way for the use of NKX2–3 as fresh biomarkers or molecular targets in potential diagnostic and therapeutic strategies for prostate cancer.

TCGA and GEO are the most commonly used high-throughput sequencing databases for cancer research. In our study, we first analyze the expression profiles of ARGs from TCGA and obtained a 17 autophagy-related prognostic genes (ATG16L1, FADD, GABARAPL2, NKX2–3, MYC, MAPK8IP1, WDR45B, MTMR14, HGS, USP10, NPC1, BIRC5, BNIP3, ATG3, RAB24, ULK3 and RUBCN) by univariate Cox regression analysis for detecting the prognosis of prostate cancer patients. Further multivariate Cox regression analysis was performed to select 6-gene prognostic signature (FADD, GABARAPL2, MYC, RAB24, RUBCN, and NPC1) and calculated the risk score of the prostate cancer patients. Our results suggested that the risk score is an independent prognostic factor and the predictive ability was improved by using the 6-gene risk signature when compared to using other clinical characteristics (age, T stage and N stage). Meanwhile, we deeply analyze the expression profiles from GEO database and obtained a total of 12 DEMs and 1073 DEGs (adjust *P* value < 0.05 and |log FC| > 1) including 2 up-regulated DEMs, 10 down-regulated DEMs, 413 up-regulated DEGs and 660 down-regulated DEGs. Then, GO function (BP, CC, and MF) analysis of DEMs and DEGs demonstrated the majority of miRNAs and genes were involved in some processes and pathways, such as cell communication, and signal transduction in BP [42, 43], extracellular space, and nucleus in CC [44, 45], transcription factor activity, extracellular matrix structural constituent in MF [46, 47], which is consistent with the previous studies on tumors. Biological pathway analysis of DEGs and DEMs showed the majority of genes and miRNAs were participated in in regulating epithelial-to-mesenchymal transition [48], PI3K-Akt signaling pathway [49], mTOR pathway [50], EGF receptor pathway [51], VEGF and VEGFR network [52], IFN-gamma pathway [53], PDGF receptor network [54], and ErbB receptor signaling network [55]. Previous studies had shown these pathways played vital roles in PCa progression.

Recently, autophagy has emerged as a potential therapeutic target for the treatment of PCa [56, 57]. The ATG3-AKT-mTOR signaling pathway is an essential regulator of autophagy in PCa [58]. ATG3 (autophagy related 3), a known critical regulator of autophagy. It has a significant effect on mediate autophagy in PCa [59, 60]. In this study, we presented a prognostic signature to PCa, which including six ARGs (NPC1, RUBCN, RAB24, MYC, GABARAPL2 and FADD). Of them, RAB24, RUBCN and GABARAPL2 had attracted our great interests. Because as shown in Fig. 6a, the above three ARGs were predicted closely linked with ATG3. However, the specific molecular mechanism by which ATG3 regulates RAB24, RUBCN and GABARAPL2 remains unclear and needs to be further verified in future studies.

Numerous studies have demonstrated that some of cancer-related miRs were implicated in autophagy regulation, miRs have been suggested as a potential element to mediate autophagy pathway in carcinomas [61]. Apart from the direct regulation between miRs and autophagy-related genes, increasing evidences indicates that autophagy is capable of regulating miR homeostasis via degrading the miR-induced silencing complexes (miRISC) [62]. ARGs are crucial for cellular processes and are directly regulated by multiple miRs [63]. A number of previous studies have demonstrated that the effects of miRs on autophagy genes and proteins were critical for cancer-related outcomes. For instance, miR 23b regulates radioresistance of pancreatic cancer cells by targeting autophagy-related 12 (ATG12) [64]. MiR-205 inhibits autophagy by targeting TP53INP1 in prostate cancer cells [65]. MiR-143 inhibits cell proliferation by targeting autophagy-related 2B (ATG2B) in non-small cell lung cancer [66]. In order to verify this relation, the dual-luciferase reporter assay is the most common method. Relative luciferase activity was measured using dual luciferase assay, and the activity of firefly luciferase was normalized with that of renilla luciferase as inner control.

In our project, we predicted target genes of 12 DEMs by FunRich software, and found that among the many autophagy-related genes, NKX2–3 was predicted to have a special correlation with miR-205 in DEMs. Some studies have indicated that miR-205 is abnormally expressed in a variety of carcinomas, and miR-205 expression levels vary in humans to function as either tumor suppressors or promoters [67]. For instance, miR-205 was down-regulated in pancreatic cancer samples, and elevated miR-205 levels inhibited pancreatic cancer cell proliferation via RUNX2 [68]. In the study of ovarian cancer, miR-205 was up-regulated in tumor tissues. Up-regulated miR-205 promoted the proliferation of ovarian cancer cells by targeting PTEN/SMAD4 [69]. In breast cancer research, the results revealed that the expression of miR-205-5p was decreased in breast cancer tissues and miR-205-5p may inhibit gemcitabine resistance in breast cancer cells via inhibition of ERp29 expression [70]. However, there are few studies on the function of miR-205 in autophagy and cancer, especially for miR-205 and NKX2–3. The mechanism of miR-205 in cancer and autophagy still needs to be further studied.

NKX2–3 is a member of the NKX family that play critical roles in regulating lymphoid organ development, tissue differentiation, and tissue-specific gene expression [71, 72]. According to available data, NKX2–3 has been demonstrated to be down-regulated in colorectal cancer, and it may contribute to the sporadic colorectal cancer by regulating the Wnt signaling pathway [73, 74]. In the study of gastrointestinal neuroendocrine carcinomas, people found that the expression of NKX2–3 in liver metastases was lower than that in primary tumor tissues, suggesting that NKX2–3 may be associated with the process of tumor metastasis [75]. NKX2–3 may also be classified as biomarker to predict the effects of primary advanced colorectal cancer patients who will undergo FOLFOX4 [76]. NKX2–3 has been studied not only in solid malignancies, but also in hematopoietic malignancies [77]. Therefore, it is very valuable to further study NKX2–3 in both solid malignancies and hematopoietic malignancies.

In our study, the expression of autophagy-related gene NKX2–3 was significantly up-regulated in prostate cancer tissues compared to the non-tumor tissues. According to univariate COX regression analysis results, NKX2–3 was found to be significantly correlated to OS in prostate cancer, the gene was identified as risk factor (HR > 1). Based on STRING analysis results, we found that the NKX2–3 was moderately related to the part of genes among the 6 autophagy-related genes prognostic signature. Then, the effect of NKX2–3 on the OS of prostate cancer was analyzed by cBioPortal database. Our data showed that patients in the altered group had a shorter overall survival time than patients in the unaltered group. Further, we analyzed the relationship between NKX2–3 and some common clinical parameters. The results showed that the lymph nodes examined number and distant metastasis rate were significantly increased in altered group, compared with the corresponding unaltered group. In order to further understand the mechanism of NKX2–3 in prostate cancer, GSEA analysis was proformed. The results showed that the high NKX2–3 expression group was correlated to biological signaling pathways, including Myc Targets V1, unfolded protein response and Myc Targets V2. As an oncogene, Myc is aberrantly expressed in the majority of types of cancer [78]. According to previous report, the dysregulation of Myc Targets has a good correlation with bladder cancer development [79]. Therefore, we infer that the NKX2–3 high expression group might be distinct molecular features, which is worthy of future exploration. Lastly, in terms of immunotherapy, PD-1 inhibition is a promising cancer immunotherapy [80]. Previous studies have confirmed that PD-1 inhibition could improve outcomes of cancer patients compared to chemotherapy [81, 82]. TMB was a potential biomarker and was defined as the total number of somatic mutations per megabase or the nonsynonymous mutations in tumor tissues, including replacement and insertion deletion mutations. It is reported that, the objective response rate of the PD-1 inhibition was higher in patients with high TMB than in patients with low TMB [83]. In our study, we found that the expression of NKX2–3 was significantly positively correlated with TMB in PCa. In addition, NKX2–3 expression was negatively correlated with that of PD-1 in PCa. Therefore, we speculated that the NKX2–3 may serve as a potential predictor for the efficacy of anti-PD-1 therapy in PCa.

In clinical work, anti-PD1 treatment has been shown to have superior therapy efficacy on multiple tumor types, but the response rate is still much lower than desired. Gaining a deeper understanding into biomarkers for predicting the anti-PD1therapy will possibly help us better decide which patients need anti-PD1 therapy and which patients do not in the future. In our study, we demonstrated that the NKX2–3 may be a potential biomarker for predicting the efficacy of anti-PD-1 therapy in PCa. On this basis, we can detect the expression level of NKX2–3 in PCa patients and develop individualized therapeutic strategies for anti-PD1 treatment. In the meantime, NKX2–3 and miR205 are inter-related, autophagy-related miRNAs were also found to be upregulated or downregulated in many cancers, and several studies point out to their potential use as biomarkers. Therefore, miRNA manipulations through using mimics or inhibitor, or other strategies, might potentially be used as efficacy indicators for cancer treatment.

## Conclusions

In short, we identified that the six ARGs expression patterns are independent predictors of OS in PCa patients. Furthermore, our results suggest that ARGs and miRNAs are inter-related. To our knowledge at present, this is the first time that the six ARGs prognostic signature and the effect of NKX2–3 on the prediction of anti-PD-1 therapy were identified in prostate cancer.

## Data Availability

The data in this study are available from the corresponding author on request.

## Abbreviations

PCa: Prostate cancer
ARG: Autophagy-related gene
OS: Overall survival
DEMs: Differentially expressed miRNAs
DEGs: Differentially expressed genes
TMB: Tumor mutation burden
PDCD1: Programmed cell death 1
